# Cutaneous Disease in the Horse Similar to Tinea Fovosa in Man

**Published:** 1883-10

**Authors:** R. Bassi

**Affiliations:** In Il Medico Veterinario, Torino


### Art. XXIX.—CUTANEOUS DISEASE IN THE HORSE SIMILAR TO TINEA FAVOSA IN MAN.

BY PROFESSOR R. BASSI.

*In Il Medico Veterinario, Torino.*

THE first cases of parasitic disease which I undertake to describe did not come to my notice in Turin until the year 1876. Before that time I happened to observe, principally in young horses imported from foreign countries, especially from England, herpes tonsurans, never the disease above referred to. My first observations were on American, French and English horses. The disease is more dreaded by horse-dealers than herpes tonsurans, as it is a more serious one whether clinically or economically considered. It usually attacks the back and side in the neighborhood of the root of the tail. It consists of an eruption of pimples which develop and run their course in three or four weeks according to the greater or lesser depth to which the inflammatory action extends or to the effects of the pressure or friction of the harness.

In the years 1877–82, I had been enabled to observe the disease in the stables of the horse merchants in Turin, Milan, Paris and London; had become convinced that it was new and entirely different from the ordinary cutaneous affections of the horse. In England it was regarded as an infection introduced with the horses imported from North America, especially Canada. On close investigation I found that the disease was propagated by the promiscuous use of harness and saddle, the parts in contact with them becoming soon covered by the peculiar eruption. The question of contagion was affirmatively settled by the results of a series of experiments which I made with the virus. Microscopical examinations resulted in the finding of a micro-organism similar to that obtained in tinea favosa of the human species.

Veterinary Surgeon Mans had already described the disease as "Pustulous exanthema," and refers to its transmission by the promiscuous use of harness and its introduction into Belgium through English horses.*

Professor Friedberger mentions it by the name of "Pustulous contagious dermatitis," and the fact that in London the American horses received the credit of its introduction.†

The clinical facts collected by the author are abridged as follows :

In the beginning of 1879 a horse merchant in Monaca imported eighteen horses directly from England. One after the other, they were all attacked by a peculiar eruption, variable in its point of origin, form and facility for diffusion by

* Annals de Mêdecine Vétérinaire, 1880.

† Wochenschrift für Thierheilkunde, 1880.

contagion. In some the eruption was observed soon after arrival; in others not until after the lapse of four weeks. The disease generally commences on the back where the girth is applied, extending behind and on the sides. The first phenomena are often attributed to a superficial contusion produced by the girth, but the rapid spread of the affection notwithstanding all possible precautions, made the error evident. The disease presents difference in respect to the extension of the area occupied; in some horses not being more than the size of a hand, whilst in others it spreads over the shoulders, sides and back. When attacked, the skin becomes painful on pressure, hot and swollen and soon round prominences appear, from the size of a pea to that of a hazel nut, either distinct and isolated or coalesced in little groups, the hairs becoming disarranged over them in little tufts. These prominences are like hard knots, somewhat limited, which penetrate the skin deeply, being more painful than itchy as in furuncle. After progressing gradually six or eight days a pustule is observed to form on their summits. On removing the necrosed tissue which forms on its surface, a dense pus appears, creamy and of healthy aspect. A scab soon forms of yellow color or brown if admixed with blood, which, implicating the surrounding hairs forms a thick covering, friable and solidly adherent. On these being detached a deep ulcer becomes visible. The base of these ulcers, of a round form reaching a remarkable depth in the skin, were surrounded by tissues in a state of inflàmmatory infiltration of a grizzly yellow color and were covered by a purulent exhudation. Their margins were easily broken down. On cessation of inflammatory action the ulcers granulated and cicatrized, this occurring generally about the fourteenth day. The general condition is seldom disturbed,

The treatment in mild cases consists in keeping the parts thoroughly clean and disinfected. If this should not prove effectual, mercurial ointment will be found useful. It is hardly necessary to insist on the precautionary measures which should bé taken to prevent the spread of the contagion.

